# Selective Adsorption of Methyl Orange and Methylene Blue by Porous Carbon Material Prepared From Potassium Citrate

**DOI:** 10.1021/acsomega.3c04124

**Published:** 2023-09-15

**Authors:** Song Wang, Jiali Dou, Tingting Zhang, Sanxi Li, Xuecheng Chen

**Affiliations:** † School of Environmental and Chemical Engineering, Shenyang University of Technology, Shenyang 110870, China; ‡ Faculty of Chemical Technology and Engineering, West Pomeranian University of Technology, Szczecin, Piastów Ave. 42, 71-065 Szczecin, Poland

## Abstract

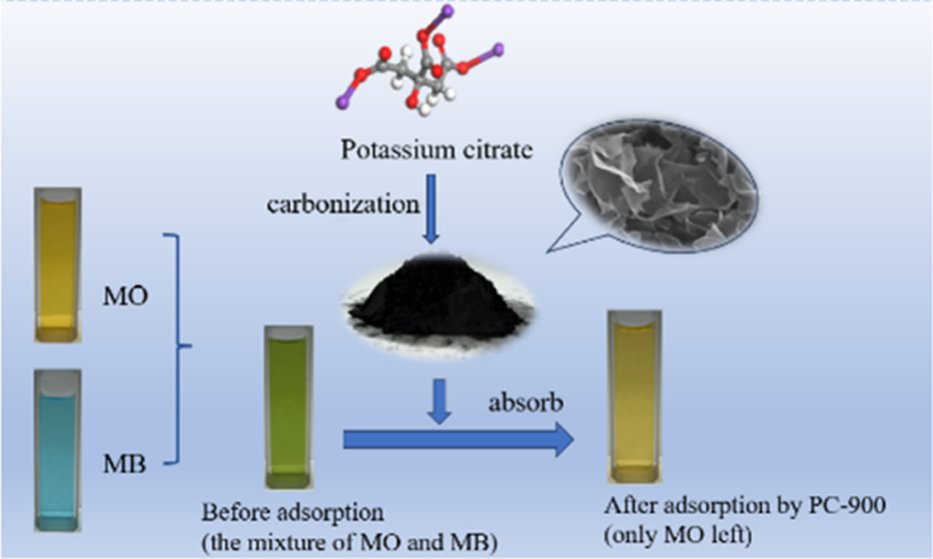

As the discharge amount of dye wastewater increases with the development of the textile printing and dyeing industries, the treatment of the dyes in the wastewater becomes more complex. The adsorption method is a commonly used method for treating dye wastewater. The adsorbent is the key factor affecting the adsorption performance. To develop a high-performance adsorbent, a porous carbon material prepared from potassium citrate by the calcination method was applied in the adsorption of dye-containing water in this study. The morphology and pore structure of the porous carbon materials were characterized by scanning electron microscopy (SEM), X-ray diffraction (XRD), and N_2_ adsorption/desorption isotherm. The porous carbon material with a specific surface area of 1436 m^2^ g^–1^, PC-900, was used as an adsorbent for the adsorption of methyl orange (MO) and methylene blue (MB). The results showed that the maximum adsorption capacity of PC-900 for MO and MB reached 927 and 1853.6 mg g^–1^, respectively. Studies on adsorption kinetics and adsorption isotherms showed that the pseudo-second-order kinetic model and the Langmuir isotherm model were more appropriate to describe the adsorption process of MO and MB by PC-900. In addition, the results of the mixed adsorption experiment of MO and MB dyes showed that PC-900 had selective adsorption for MB.

## 1 Introduction

With the mushroom growth of the printing and dyeing industry, the use of dyes is becoming more and more extensive because dyes can give materials such as fiber and leather beautiful colors and give people a good visual effect.^1,2^ The development of the printing and dyeing industry leads to the production of a large amount of wastewater.^3,4^ The discharge of wastewater containing organic pollutants, which contain a high concentration of dyes, is one of the reasons for water pollution. The existence of dyes in wastewater can not only destroy the environment but also lead to nasty damage to the organism and human body, which can cause the deterioration of the ecosystem and hinder the sustainable development of human society.^5,6^ Since people have realized the importance of environmental safety, it is necessary to explore an efficient, low-cost, and pollution-free measure to remove dyes from wastewater.^7,8^

A variety of methods have been developed to treat dye wastewater such as flocculation sedimentation, ion exchange, ultrafiltration, dialysis, chemical oxidation, photooxidation, electrolysis, adsorption, and other methods.^9−12^ Coagulation sedimentation, adsorption, chemical oxidation, and biological treatment are the main methods that were applied in the current industrial of dye wastewater treatment.^13^ Among these methods, adsorption is the most common water treatment method, which relies on adsorbents with a high specific surface area and large adsorption capacity to remove pollutants from water.^14,15^ During the practical application, the adsorption method shows advantages in the aspect of a simple and fast treatment procedure.^16^ The adsorbent is the pivotal factor in determining the adsorption treatment performance.^17^ A large number of adsorbents such as metal–organic frameworks (MOF), self-assembled nanostructures, porous polymer, layered double hydroxide, zeolite, and porous carbon have been developed for dye adsorption.^18−23^ Duo et al. prepared an iron-based metal–organic framework (MOF-235) to remove the Congo red (CR) and lemon yellow (LY) in the water.^18^ The adsorption capacities of the obtained MOF-235 for CR and LY were 1131 and 209 mg g^–1^, respectively. Jeyarani et al. synthesized WO_3_ nanostructures with variable crystal phases, WO_3_(H_2_O)_0.5_ and (NH_4_)_0.33_WO_3_, which can self-assemble into structures such as nanoplates and nanospheres.^23^ The nanospheres of the WO_3_ nanostructure exhibited a maximum adsorption capacity of 116 mg g^–1^ for MB. Jiang et al. developed a novel β-cyclodextrin-based porous polymer material by cross-linking β-cyclodextrin with tetrafluoroterephthalonitrile followed by the modification with ethanolamine.^19^ The porous polymer material exhibited high adsorption capacities of 602 and 1085 mg g^–1^ for MO and MB, respectively. Compared with these adsorbents, porous carbon material has the advantage of low preparation cost. Numerous biomass waste or biomass derivatives can be used to prepare porous carbon material.^24,25^ Liu et al. obtained a porous carbon from eggplants, EPCM, which had a hierarchical porous structure with a total pore volume of 0.6379 cm^3^ g^–1^ and a specific surface area of 516 m^2^ g^–1^. The adsorption capacity of EPCM for rhodamine B reached 263 mg g^–1^.^26^ Using litchi peel as the raw material, Wu et al. prepared a porous carbon material, HLP850, with a high specific surface area of 1006 m^2^ g^–1^. The adsorption capacities of CR and malachite green were 404.4 and 2468 mg g^–1^ when HLP850 was devoted to the management of dye wastewater.^27^ Developing high-performance porous carbon materials has become a significant task in the realm of adsorbents.

Potassium citrate is a biomass derivate that is synthesized from citric acid extracted from the juice of lemons, limes, etc. With the maturity of fermentation technology for preparing citric acid, the utilization of potassium citrate has also attracted increasing attention.^28^ Porous carbon materials prepared from potassium citrate have shown promising application potential in fields such as supercapacitors, carbon capture and storage, and adsorbents.^28−30^ Herein, potassium citrate was used to obtain porous carbon materials by a calcination method. The influence of the calcination temperatures on the adsorption capacity of the prepared porous carbon samples was compared. The porous carbon material with the best adsorption performance was applied in the study of the adsorption kinetics, adsorption isotherms, and selective adsorption in MO and MB dye mixtures.

## 2 Materials and Methods

### 2.1 Materials

Potassium citrate monohydrate (AR) was purchased from Tianjin Da Mao, China. MO (AR) and MB (AR) were purchased from Tianjin Zhi Yuan, China. All chemicals were used directly with the purification step.

### 2.2 Preparation of Porous Carbon Materials

First, the potassium citrate samples were calcined at 250 °C for 30 min under a N_2_ atmosphere in a vacuum tube furnace. Second, the samples were sequentially heated to the selected carbonization temperatures (700, 800, and 900 °C) at a heating rate of 3 °C min^–1^ and maintained at the established temperature for 60 min. Third, the carbonized samples were washed with distilled water until the acid–base was neutral after the device was at room temperature. The finally obtained samples were dried in a vacuum oven at 120 °C for 6 h, which were named PC-*X* (*X* indicated the carbonization temperatures, including 700, 800, and 900 °C).

### 2.3 Characterization Methods

The thermal decomposition behavior of the material was detected by thermogravimetric analysis (TGA, Q50). The test conditions were heated from room temperature to 900 °C at a heating rate of 10 °C min^–1^ in a N_2_ atmosphere. The morphology of samples was characterized by scanning electron microscopy (SEM, Hitachi SU8010, Japan). The crystalline structure of samples was recorded by X-ray diffraction (XRD, Rigaku Miniflex600, Japan). The functional groups of the samples were recorded by Fourier transform infrared spectra (FTIR, IR Prestige-21, Japan). The pore structure of samples was analyzed by the nitrogen adsorption/desorption technique (Kubo X1000, China). The determination of the ζ potential of the sample was performed with a microelectrophoresis instrument (JS94H, China) using distilled water as a solvent. UV–vis examination data were obtained on an Analytical Tu-1810 spectrophotometer.

### 2.4 Adsorption Experiments

A certain mass of the adsorbent was appended to a conical flask containing a certain volume of dye solution with a certain concentration. The adsorption was completed by shaking the conical flask at a certain temperature and time. After the adsorption, the adsorbent was removed by filtration, and the absorbance was measured at the maximal absorption wavelength of MO (λ = 464 nm) and MB (λ = 665 nm) by a UV–vis spectrophotometer. The amount of MO or MB adsorption capacities at equilibrium *q*_e_ (mg g^–1^) and the removal rate *r* (%) were calculated by the following equations^31−33^
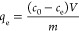
1
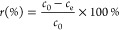
2where *c*_0_ (mg L^–1^) is the initial concentration of the dye solution, *c*_e_ (mg L^–1^) is the concentration of the dye solution at adsorption equilibrium, *V* (mL) is the initial volume of the dye solution, and *m* (g) is the dosage of the adsorbent.

The detailed parameters related to the adsorption experiments are presented as follows:

Influence of pH on adsorption capacity: The pH of the dye solutions was adjusted to 2, 4, 6, 8, and 10 by a 0.1 mol L^–1^ hydrochloric acid or potassium hydroxide solution. Then, 10 mg of the absorbent was added into 100 mL of a 100 mg L^–1^ MO solution or 100 mL of a 100 mg L^–1^ MB solution to adsorb 10 h at 35 °C, respectively. After adsorption, the supernatant was separated by an aqueous poly(ether sulfone) filter membrane with a pore size of 0.22 μm, and the absorbance of the supernatant was measured.

Study on the adsorption kinetics: 10 mg of the adsorbent was added into a series of a 100 mg L^–1^ MO solution or 200 mL of a 100 mg L^–1^ MB solution to adsorb for 5–600 min at 35 °C, respectively. Then, the corresponding kinetic models were fitted with the obtained data.

Study of the adsorption isotherms: A suite of dye solutions with a concentration ranging from 10 to 200 mg L^–1^ was prepared. Then, 10 mg of the adsorbent was added into the series of 100 mL of the MO solution or 200 mL of the MB solution to adsorb for 10 h at 35 °C, respectively. Finally, the corresponding isotherm models were fitted by the obtained data.

Study of selective adsorption: The same volume of the MO solution and the MB solution (50 mL) of the same concentration (200 mg L^–1^) were evenly mixed to get a mixed dye solution. Ten milligrams of the adsorbent was appended to the 100 mL mixed solution to adsorb for 10 h at 35 °C. The changes in the absorbance of the mixed dye solution were recorded.

Study on regeneration performance: The regeneration experiments of the adsorbent after the adsorption were carried out according to the methods reported in the literature.^10,14^

## 3 Results and Discussion

### 3.1 Structural Characterization

**Figure 1:**
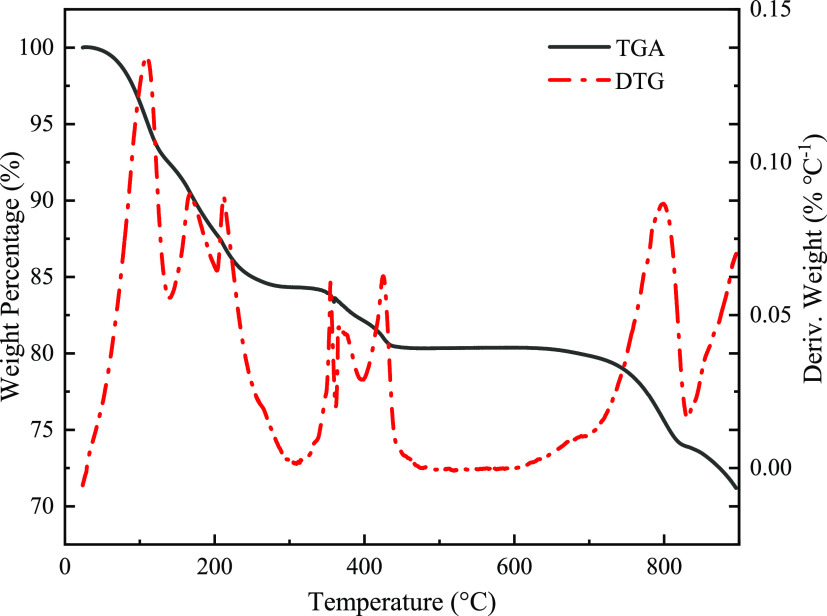
TGA and DTG curves of potassium citrate.

To determine the thermal decomposition behavior of potassium citrate, we implemented thermogravimetric analysis (TGA). The result is shown in Figure 1. The residue mass rate was about 70% when the temperature reached 900 °C. Meanwhile, the decomposition of potassium citrate could be split into three stages on the basis of the DTG curve. The first decomposition stage, which occurred below 300 °C, was caused by the decomposition of some carboxyl groups in potassium citrate and crystalline water, resulting in a mass loss of about 15%. In the second decomposition stage, potassium citrate decomposed into potassium carbonate and carbon. The decomposition reaction occurred in the range 300–500 °C, resulting in a mass loss of about 5%. The reaction above 500 °C belonged to the third decomposition stage, which mainly includes K_2_CO_3_ → CO_2_ + K_2_O and K_2_O + C → 2K + CO. The mass loss caused by these two reactions was about 10%.^29^ A large amount of CO and CO_2_ produced in the decomposition process of potassium citrate made it form an abundant porous structure, which was why activated carbon materials prepared with potassium citrate could be used as adsorbents. The TGA curve showed that the thermal decomposition behavior of potassium citrate was intricate. Particularly, when the carbonization temperature was higher than 700 °C, potassium citrate did not stop its thermal decomposition. As a result, when the carbonization temperature was higher than 700 °C, the structure of the carbon residue was different. Therefore, the structure and adsorption properties of the porous carbon materials prepared from potassium citrate at different carbonization temperatures must be first investigated.

**Figure 2:**
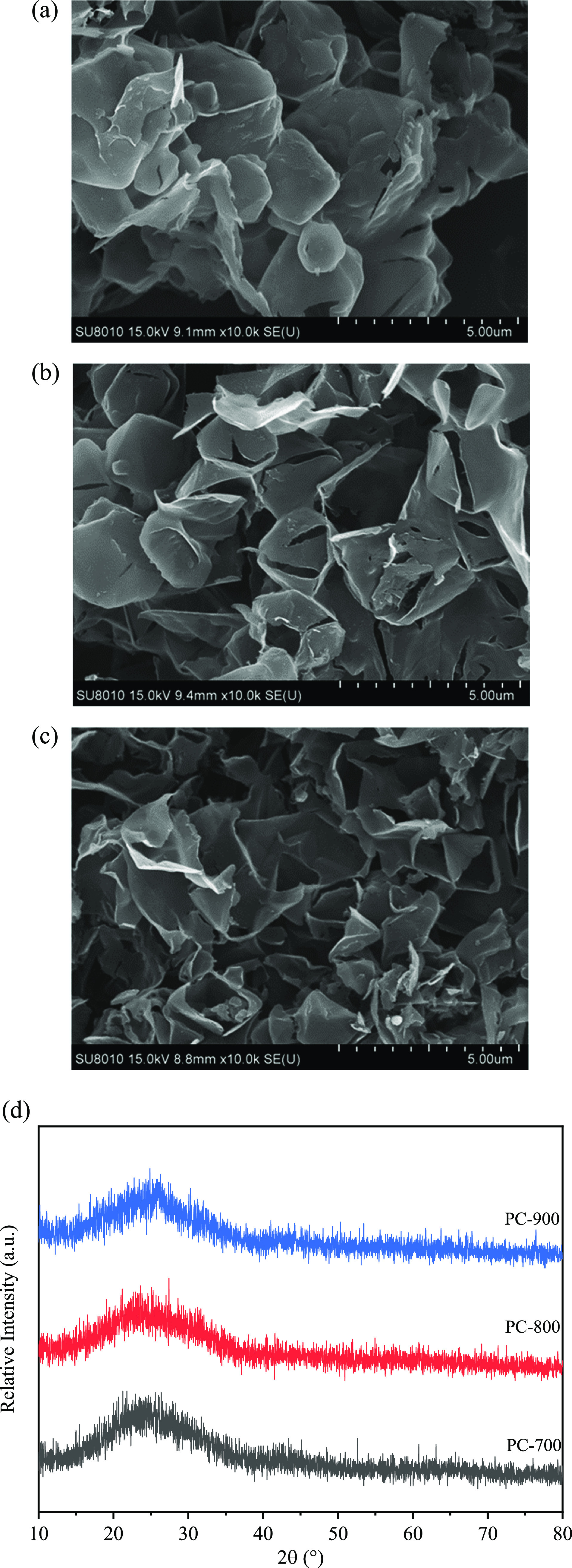
SEM images of (a) PC-700, (b) PC-800, and (c) PC-900. (d) XRD profiles of PC-700, PC-800, and PC-900.

To study the impact of the carbonization temperature on the microstructure of porous carbon materials, the morphology of the prepared samples is shown in Figure 2. PC-700 is mainly the aggregation of irregular hollow cavities, which are composed of thin carbon sheets. As the carbonization temperature increased to 800 °C, more and more big holes could be observed on the surface of irregular hollow cavities. However, when the carbonization temperature was added up to 900 °C, the original irregular hollow cavities were destroyed completely, and a large number of irregular holes were formed. The morphology of PC-900 seems like the aggregation of an irregular thin carbon sheet. The morphology investigation results revealed the different microstructures of the obtained porous carbon samples. To determine the crystalline structure of the prepared carbon materials, XRD patterns of the three carbon materials were recorded, which are shown in Figure 2(d). The prepared carbon materials have similar diffraction patterns. A broad diffraction peak at 25° could be observed in the XRD patterns of the three carbon materials, which indicates the existence of the amorphous carbon.^26,27^

**Figure 3:**
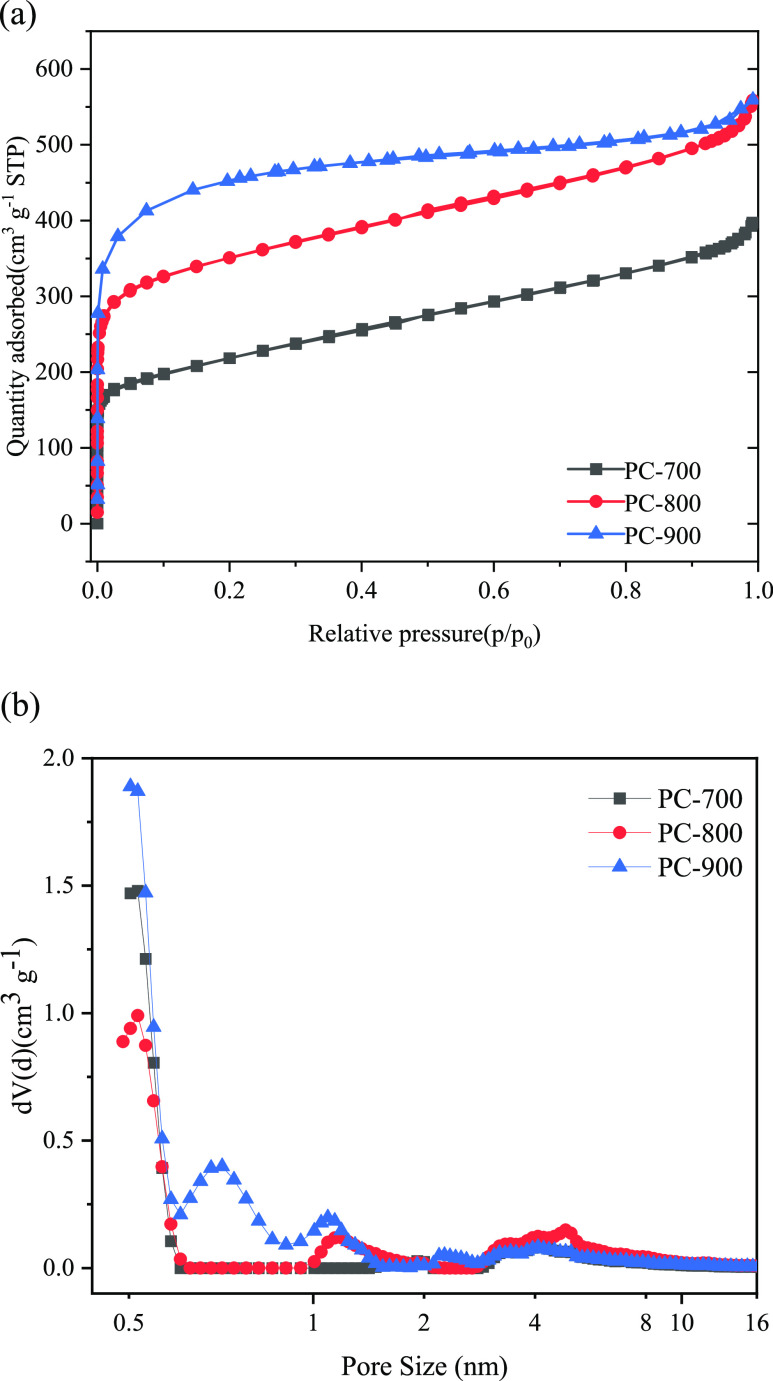
(a) N_2_ adsorption/desorption isotherms of PC-700, PC-800, and PC-900; (b) pore size distribution curves of PC-700, PC-800, PC-900.

The specific surface area and the pore structures of the prepared porous carbon materials were tested by the N_2_ adsorption/desorption method. As shown in Figure 3(a), PC-700, PC-800, and PC-900 showed typical type-I isotherms, indicating the presence of micropores in their structures.^30^ At a low relative pressure (*P*/*P*_0_ < 0.05), the N_2_ isotherm exhibited a sharp increase, which meant abundant micropores in the carbon material. Besides, compared with PC-700 and PC-800, PC-900 showed a higher N_2_ adsorption capacity, which meant that there were more microporous structures in PC-900. Furthermore, an apparent tail was shown at *P*/*P*_0_ ≥ 0.9, indicating the presence of a spot of macropores in the porous carbon sample. The specific surface areas of the PC-700, PC-800, and PC-900 carbon materials were 775, 1291, and 1436 m^2^g^–1^, respectively. In summary, the carbon material prepared from potassium citrate had a porous structure with plenty of pore structures.

The pore size distribution curves of PC-700, PC-800, and PC-900 are shown in Figure 3(b). It could be seen that most of the aperture of PC-700 was in the range of 2–10 nm, while most of the apertures of PC-800 and PC-900 were in the range of 0.5–10 nm, which meant that compared with PC-700, PC-800 and PC-900 had a wider pore size distribution. The total pore volumes of PC-700, PC-800, and PC-900 were 0.615, 0.865, and 0.956 cm^3^ g^–1^, respectively. The different pore size distributions and total pore volumes of PC-700, PC-800, and PC-900 can lead to the different adsorption properties of PC-700, PC-800, and PC-900.

### 3.2 Adsorption of Dyes by the Prepared Porous Carbon Material

#### 3.2.1 Comparison of the Adsorption Capacity of the Prepared Porous Carbon Materials

**Figure 4:**
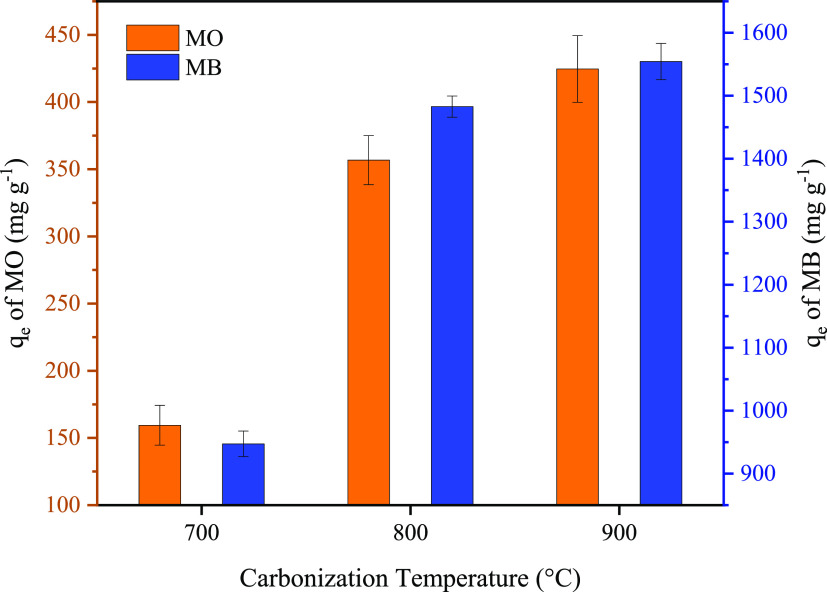
Adsorption performance of MO and MB. (10 mg of PC-700, PC-800, and PC-900 was put into 100 mL of a 100 mg L ^–1^ MO solution, and 10 mg of PC-700, PC-800, and PC-900 was put into 200 mL of a 100 mg L^–1^ MB solution).

Figure 4 shows the adsorption properties of porous carbon materials prepared at different carbonization temperatures for MO and MB. The maximal adsorption capacities on MO of PC-700, PC-800, and PC-900 were 159.4, 356.8, and 424.6 mg g^–1^, respectively. The maximal adsorption capacities on the MB of PC-700, PC-800, and PC-900 were 947.2, 1482.8, and 1554.4 mg g^–1^, respectively. Due to the highest BET-specific surface area of PC-900, which can provide more area to adsorb dyes, PC-900 showed the best performance on the adsorption of MO and MB among the prepared porous carbon materials. To confirm the MO and MB adsorption by PC-900, the functional groups of MO, PC-900, and PC-900 after the adsorption experiment were determined by the FTIR test, and the corresponding results are presented in Figure S1. The bands at 1602 and 1591 cm^–1^ in the FTIR spectra of PC-900 after the MO adsorption experiment and the MB adsorption experiment, respectively, indicate the successful adsorption of MO and MB by PC-900.^34,35^ To demonstrate the good adsorption performance of PC-900, the adsorption capacity of MO and MB by PC-900 was compared with those of other reported porous carbon materials (Table 1). PC-900 showed relatively good adsorption capacity for MO and MB among the listed porous carbon materials. Therefore, PC-900 was used in the subsequent adsorption experiments.

**Table 1:** Comparison of the Adsorption Performance of PC-900 with that of the Reported Porous Carbon Materials

adsorbent	*q*_m_ (mg g^–1^)	dye	reference
PC-900	1853.6	MB	this paper
ginger straw waste-derived porous carbons	98.5	MB	[^17^]
nitrogen-doped and hierarchically porous carbon derived from spent coffee	201.7	MB	[^22^]
three-dimensional hierarchical porous carbon derived from jujube	925.9	MB	[^36^]
3D porous carbon from food waste	12.8	MB	[^37^]
PC-900	927	MO	this paper
LDH-decorated porous carbon	412.8	MO	[^20^]
N-doped porous carbon by pyrolyzing ZIF-8	32.7	MO	[^38^]
longan seed-derived activated carbon	464.7	MO	[^39^]
chondrus crispus-activated carbon	49.5	MO	[^40^]
abundant cilantro-derived activated carbon	467.3	MO	[^41^]

#### 3.3.2 Influence of the pH Value

**Figure 5:**
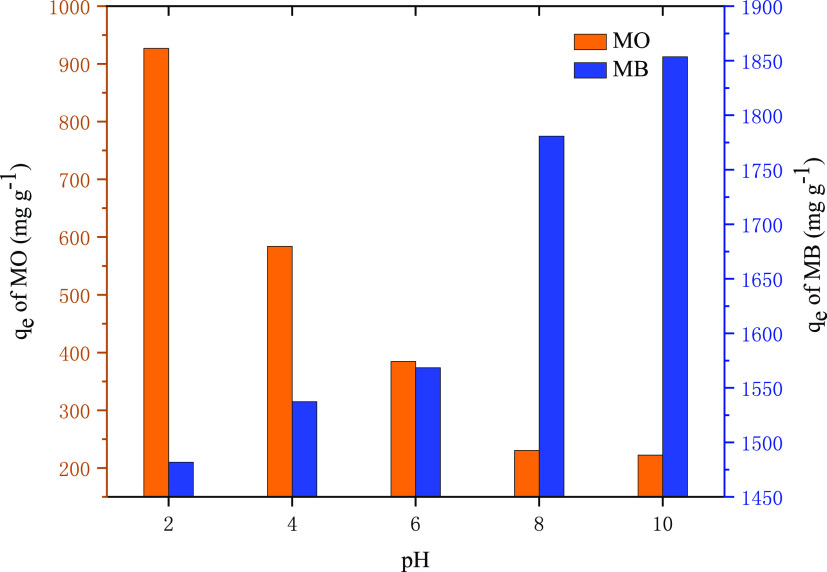
Effect of the Initial pH value on the adsorption performance of MO and MB.

The pH value of the dye solution is a significant factor affecting the adsorption behavior of the adsorbent because the pH value of the solution not only affects the surface charge of the adsorbent but also affects the ionization state of the adsorbent.^35,42^ Therefore, the influence of the dye solution pH value on the MO and MB adsorption capacity of PC-900 was investigated; the result is shown in Figure 5. Along with the pH value changing from 2 to 10, the adsorption capacity on the MO of PC-900 decreased from 927.0 to 222.4 mg g^–1^. However, along with the pH value increasing from 2 to 10, the adsorption capacity on MB of PC-900 increased from 1499.8 to 1853.6 mg g^–1^. These phenomena were related to the interaction between the molecular structure and the charge of the adsorbent.^43^ In a neutral environment, the zeta potential of PC-900 was −33.04 mv, and the polarity of the PC-900 particles was negative. When pH was lower than 7, H^+^ protonated the surface of the adsorbent, thus enhancing the electrostatic attraction between the adsorbent and anionic dye (MO), resulting in the increase of the adsorption capacity of the adsorbent to MO. On the contrary, when pH was higher than 7, OH^–^ can produce competitive adsorption with anionic dye MO, contributing to a descent in the adsorption capacity on MO of the adsorbent. The influence of the pH value on the removal of cationic dyes (MB) is the opposite.^34,44−46^

#### 3.3.3 Adsorption Kinetics and Adsorption Isotherms

The adsorption process is generally considered to consist of three stages.^47^ The first stage is the film diffusion stage, which means that the adsorbent diffuses from the liquid phase to the surface of the adsorbent. The second stage is the intraparticle diffusion stage, which means that the adsorbent diffuses into the adsorbent through fine pores. In the third stage, the adsorbent is adsorbed on the inner surface of the adsorbent. Generally, the third adsorption process is very rapid, and the adsorption rate depends mainly on the first two stages. Pseudo-first-order and pseudo-second-order kinetic models are the most commonly used kinetic models. The pseudo-first-order kinetic model supposes that the adsorption rate is related to the number of adsorption sites, and the formula is as follows^48^
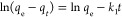
3where *q_t_* (mg g^–1^) is the adsorption capacity at *t* moment and *k*_1_ (min^–1^) is the pseudo-first-order kinetics adsorption rate constant.

The pseudo-second-order kinetic model assumes that the adsorption rate is affected by the chemisorption mechanism, and the formula is as follows^38^
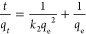
4where *k*_2_ (g min^–1^ mg^–1^) is the pseudo-second-order kinetics adsorption rate constant.

**Figure 6:**
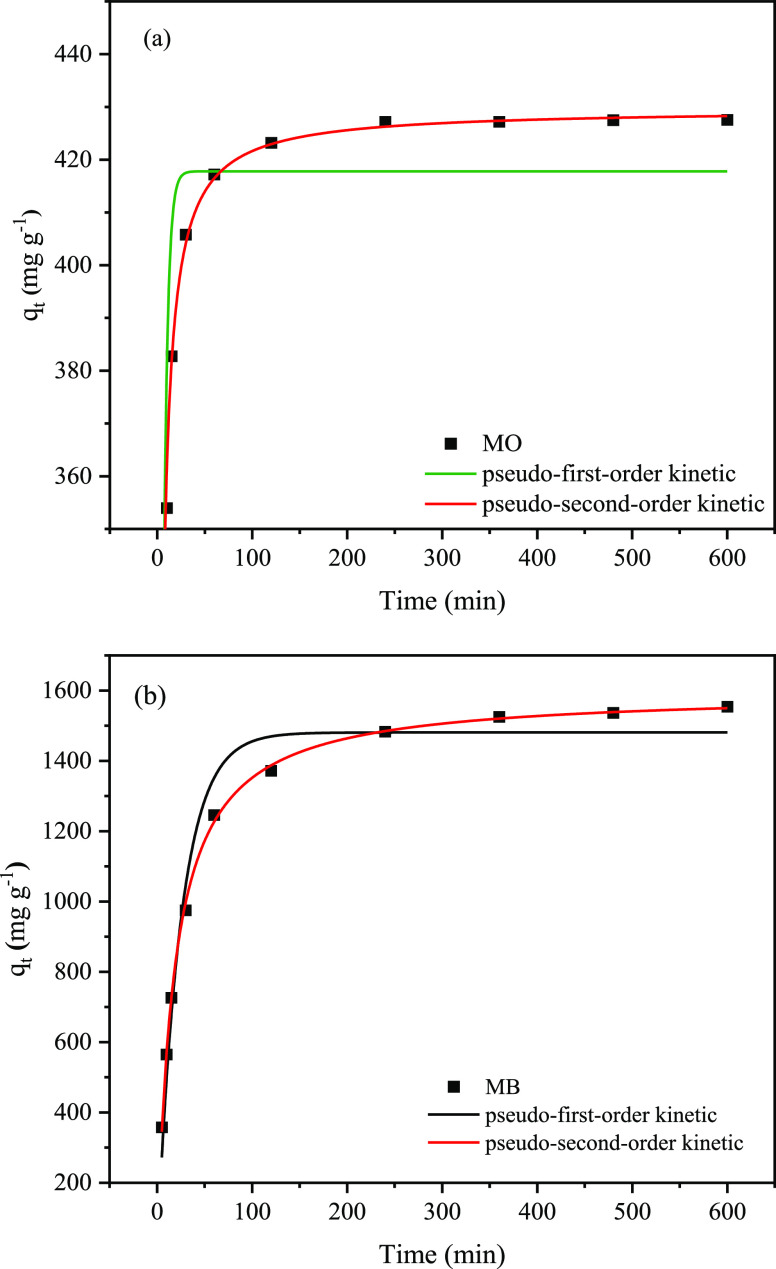
Kinetic fitting of (a) MO and (b) MB.

Figure 6 shows the relationship between the adsorption capacities of MO and MB and the adsorption time of PC-900. The color change of the MB and MO solution before and after adsorption is shown in Figure S2. The pseudo-first-order and pseudo-second-order kinetic fitting results of MO and MB are also presented in Figure 6. The pertinent fitting data are listed in Table 2. The data indicated that the *q*_e_ obtained by using the pseudo-second-order kinetic model was closer to the actual data, and the *R*^2^ obtained from the pseudo-second-order kinetic model was higher than that obtained from the pseudo-first-order kinetic model. Therefore, the adsorption kinetics of MO and MB were more consistent with the pseudo-second-order model, which indicated that the adsorption processes of MO and MB were mainly determined by the chemisorption mechanism.^49,50^

**Table 2:** Kinetic Fitting Parameters of MO and MB

			pseudo-first-order			pseudo-second-order		
adsorbate	*C*_0_(mg L^–1^)	*Q*_e,exp_(mg g^–1^)	*Q*_e,cal_(mg g^–1^)	*k*_1_ (min^–1^)	*R*^2^	*Q*_e,cal_(mg g^–1^)	*k*_2_(g mg^–1^ min^–1^)	*R*^2^
MO	100	427.58	417.80	0.24	0.8295	427.5	0.027	0.993
MB	100	1554.32	1480.78	0.041	0.9719	1570.1	0.000031	0.998

Adsorption isotherms are mainly used to analyze the adsorption behaviors between the adsorbent and the adsorbate. Many adsorption isotherms have been proposed from different perspectives; the commonly used models include the Langmuir adsorption isotherm model, the Freundlich adsorption isotherm model, and the Temkin adsorption isotherm model.^21,33,36,51−53^ The Langmuir isotherm is on account of the monolayer adsorption theory; that is, there are a certain number of adsorption sites on the adsorbent surface, and each site can adsorb a molecule, so the surface adsorption is a monolayer. The formula is as follows:
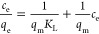
5where *q*_m_ (mg g^–1^) is the Langmuir monolayer adsorption capacity and *K*_L_(L mg^–1^) is the Langmuir adsorption constant.

The Freundlich isotherm is a nonlinear equation used to describe heterogeneous surface adsorption. The formula is as follows:
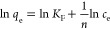
6where *K*_F_ represents the Freundlich adsorption capacity constant and *n* represents the Freundlich adsorption intensity constant.

The Temkin isotherm is based on the indirect interaction between the adsorbent and the adsorbate, and the formula is as follows:
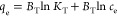
7where , *K*_T_ (L mg^–1^) is the maximum adsorption equilibrium constant, *b*_T_ is the Temkin constant, *T*(K) is the thermodynamic temperature, and *R* (8.314 J mol^–1^ K^–1^) is the universal gas constant.

**Figure 7:**
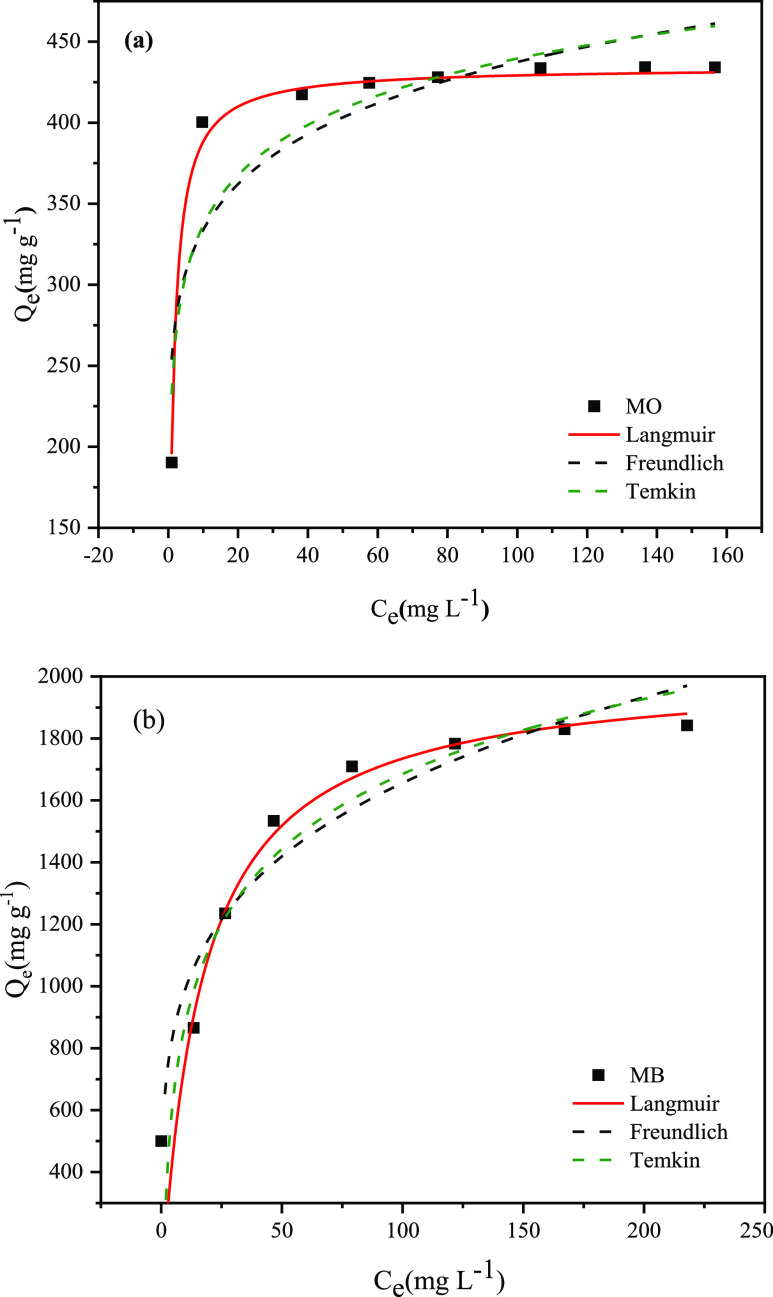
Equilibrium adsorption isotherms of (a) MO and (b) MB.

Figure 7 shows the adsorption capacities of PC-900 on MO and MB at various initial concentrations, which were fitted by the Langmuir isotherm, Freundlich isotherm, and Temkin isotherm models. The detailed fitting arguments are listed in Table 3. The data indicated that the *q*_m_ obtained by the Langmuir model fitting was closer to the actual adsorption capacity, and the *R*^2^ obtained by the Langmuir model was higher than 0.99, which is the highest *R*^2^ value among the *R*^2^ values obtained from the three models. Therefore, the isothermal adsorption experiment results showed that MO and MB were adsorbed on the surface of PC-900 in the form of monolayer adsorption.

**Table 3:** Adsorption Isotherm Fitting Parameters of MO and MB

		adsorbate	
isotherm	parameter	MO	MB
Langmuir	*q*_m_ (mg g^–1^)	446.43	1929.38
	*K*_L_ (L mg^–1^)	0.695	0.0965
	*R*^2^	0.99468	0.99232
Freundlich	1/*n*	0.14959	0.25904
	*K*_F_ [(mg g^–1^)(L mg^–1^)^1/*n*^]	223.55	504.72
	*R*^2^	0.78154	0.88318
Temkin	*B*_T_	44.723	349.338
	*K*_T_ (L mg^–1^)	185.638	1.2447
	*R*^2^	0.84467	0.82748

#### 3.3.4 Adsorption Selectivity

**Figure 8:**
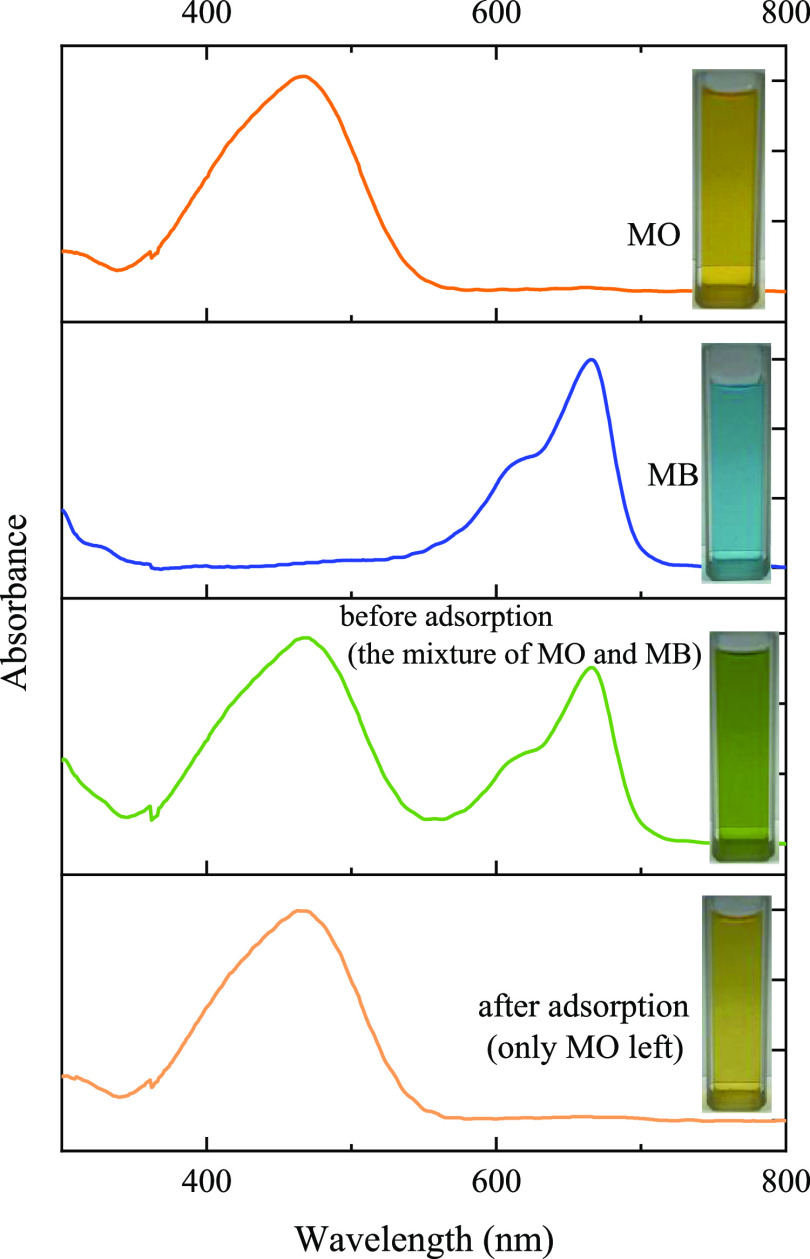
Absorbance curve and the picture of MO, MB, the mixture of MO and MB before adsorption, and only MO left after adsorption. The photograph is courtesy of “Jiali Dou”. Copyright 2022.

The selective adsorption property of PC-900 was explored in the compounds of MO and MB to clearly distinguish the difference in the adsorption sequence between MO and MB. The color and dye characteristic absorption peaks of the mixed solution after adsorption were compared with those before adsorption, as shown in Figure 8. The color of the MO and MB were orange and blue, with the adsorption peaks at 464 and 665 nm, respectively. The color of the mixture of MO and MB was green, with the appearance of the adsorption peaks at 464 and 665 nm. After the adsorption by PC-900, the color of the mixture turned orange and only the adsorption peak at 464 nm can be observed, which indicated that MB had been almost removed from the mixture and only MO was left in the mixture. The above results intuitively indicated that when MO and MB simultaneously existed in the water, PC-900 had preferential adsorption for MB, which indicated a potential application of PC-900 in the separation of the mixed dyes.^37,54^

#### 3.3.5 Regeneration Performance Study

**Figure 9:**
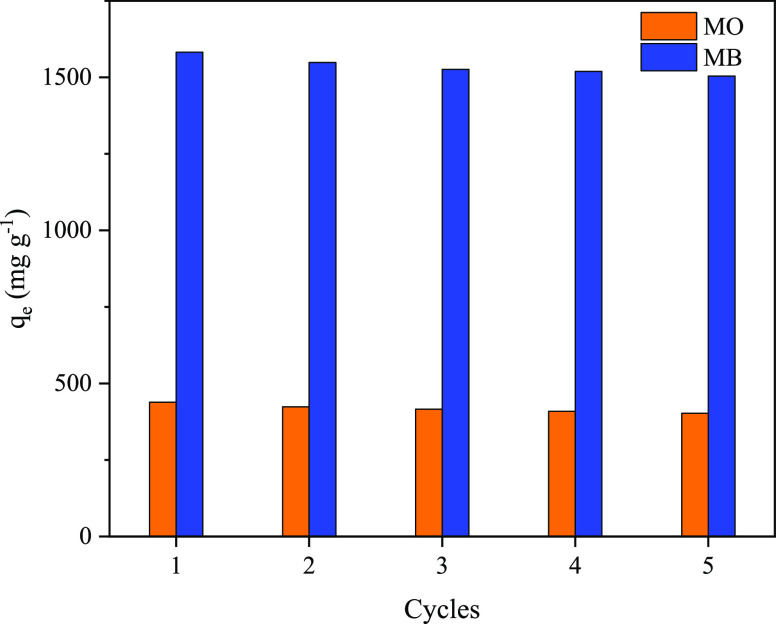
Reusability of PC-900 for the adsorption of MO and MB.

Reusability is an important property of adsorbents. The reusability experiment result of the PC-900 is shown in Figure 9. After five times of reuse, the adsorption capacity of PC-900 for MO and MB can still reach 402.6 and 1502.9 mg g^–1^, which decreased by less than 8.2 and 5.1%, respectively, compared with the initial adsorption capacity. This reusability performance study indicated that PC-900 has good reuse performance.

## 4 Conclusions

Three porous carbon materials were prepared from potassium citrate by calcination at different temperatures. PC-900, which was obtained at a calcination temperature of 900 °C, has the highest specific surface area of 1291 m^2^ g^–1^ among the prepared porous carbon materials and exhibited the best adsorption performance. The maximal adsorption capacities on the MO and MB of the PC-900 reached 927.0 and 1853.6 mg g^–1^, respectively. The adsorption kinetics and adsorption isotherm investigation on the MO and MB adsorption by PC-900 indicated that the pseudo-second-order kinetic model (chemisorption) and the Langmuir isotherm model (monolayer adsorption) were suitable to describe the adsorption behavior of PC-900. The selective adsorption experiment results indicated that PC-900 preferentially adsorbs MB in the simultaneous presence of MO and MB. Therefore, potassium citrate-derived porous carbon material is a high-performance adsorbent for the removal of dyes.

## Supporting Information Available

FTIR curves for the adsorbent and the adsorbent after the adsorption and color change of the MB and MO solution before and after adsorption (PDF)
