# Supplementary material for: Selective Adsorption of Methyl Orange and Methylene Blue by Porous Carbon Material Prepared From Potassium Citrate
Source: ACS Omega. 2023 Sep 15;8(38):35024–33. doi: 10.1021/acsomega.3c04124 (PMC10535257; doi:10.1021/acsomega.3c04124)
Supplement: Supplementary file 1 — ao3c04124_si_001.pdf [file ao3c04124_si_001.pdf]

# Supporting Information

## Selective adsorption of methyl orange and methylene blue by porous carbon material prepared from potassium citrate

Song Wang[a], Jiali Dou[a], Tingting Zhang[a], Sanxi Li[a], Xuecheng Chen[a,b*]

[a] School of Environmental and Chemical Engineering, Shenyang University of Technology, Shenyang 110870, China.

[b] Faculty of Chemical Technology and Engineering, West Pomeranian University of Technology, Szczecin, Piastów Ave. 42, 71-065 Szczecin, Poland.

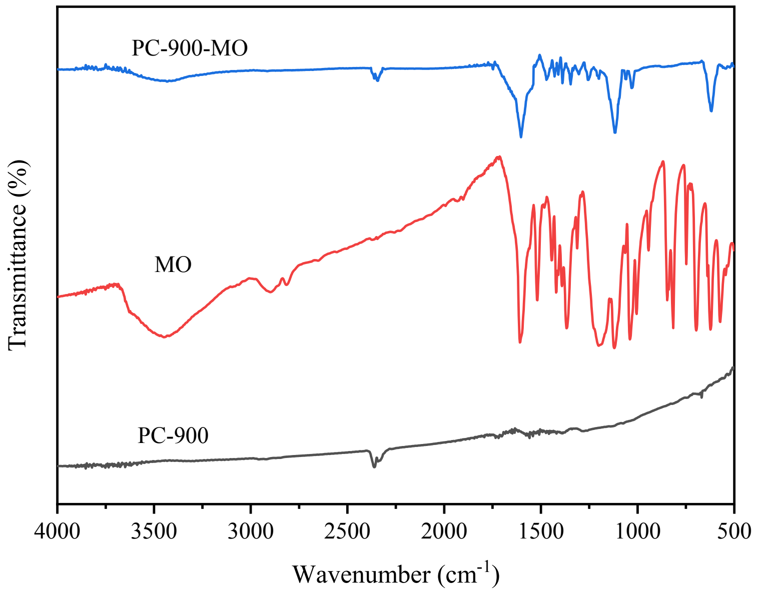


(a)

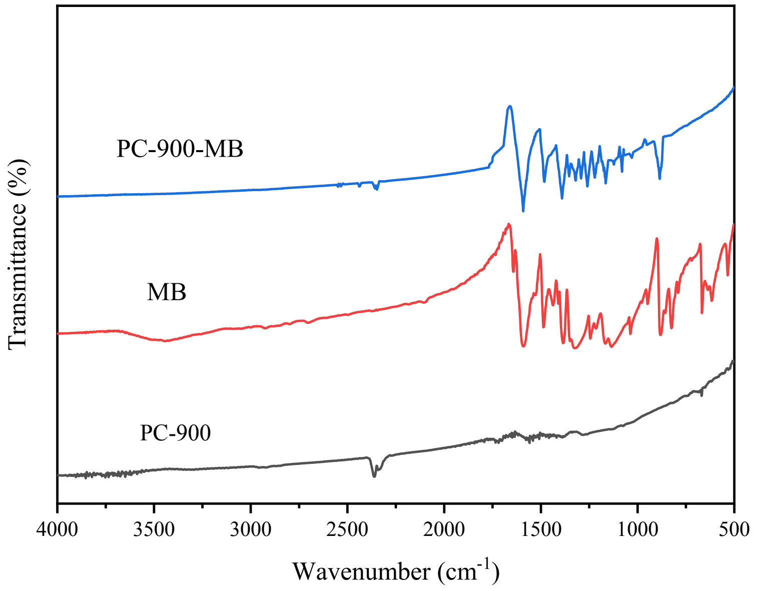


(b)

Figure S 1. (a) FTIR curves of PC-900, MO, and PC-900-MO (PC-900 after the adsorption of MO); and (b) FTIR curves of PC-900, MB, PC-900-MB (PC-900 after the adsorption of MB).

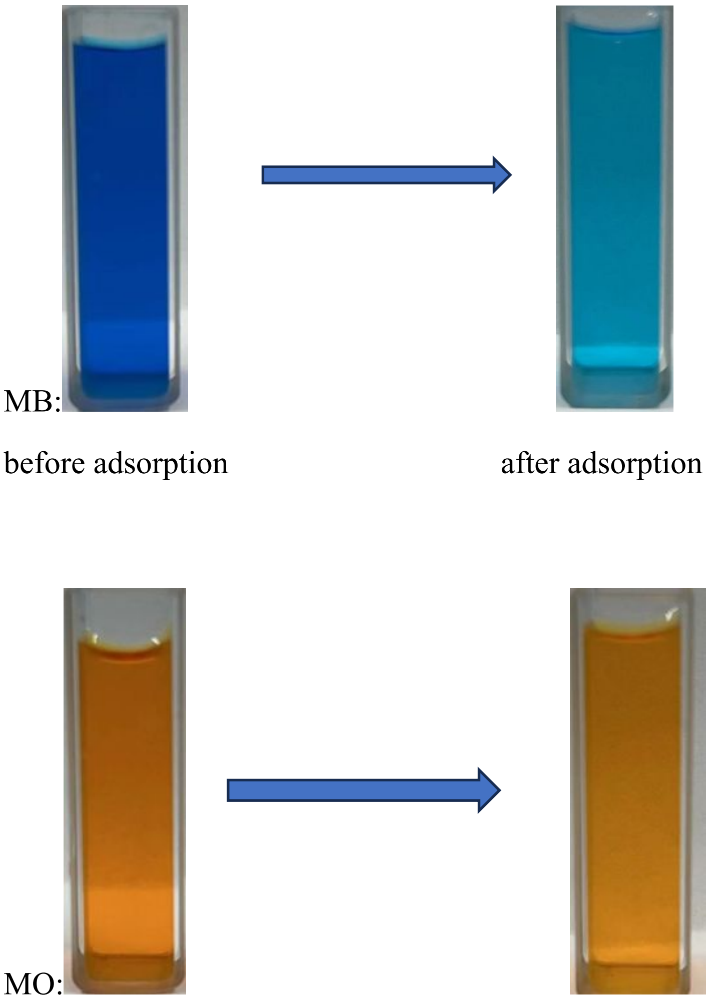

MB: before adsorption → after adsorption

MO: before adsorption → after adsorption

Figure S 2. The color change of the MB and MO solution before and after adsorption
